# Shame and Self-Esteem: A Meta-Analysis

**DOI:** 10.5964/ejop.2115

**Published:** 2021-05-31

**Authors:** Yohanes Budiarto, Avin Fadilla Helmi

**Affiliations:** a Faculty of Psychology, Universitas Gadjah Mada, Yogyakarta, Indonesia; b Faculty of Psychology, Universitas Tarumanagara, Jakarta, Indonesia; University of Wroclaw, Wroclaw, Poland

**Keywords:** shame, self-esteem, meta-analysis, meta-regression, publication bias

## Abstract

Scholars agree that shame has many effects related to psychological functioning declines, and one among others is the fluctuation of self-esteem. However, the association between shame and self-esteem requires further studies. Heterogeneity studies due to different measurements, various sample characteristics, and potential missing research findings may result in uncertain conclusions. This study aimed to explore the relationship between shame and self-esteem by meta-analysis to come up with evidence of heterogeneity and publication bias of the study. Eighteen studies from the initial 235 articles involving the term shame and self-esteem were studied using the random-effects model. A total of 578 samples were included in the study. The overall effect size estimate between shame and self-esteem (r = −.64) indicates that shame correlates negatively with self-esteem and is large effect size. The result showed that heterogeneity study was found (I² = 95.093%). The Meta-regression showed that age moderated the relationship between shame and self-esteem (p = .002), while clinical sample characteristics (p = .232) and study quality (p = .184) did not affect the overall effect size.

Self-esteem is a psychological trait that is very well known and very well studied and explained by Branden (1994) as a person's belief in their worthiness to be rejoicing and able to cope with and handle everyday life issues. Self-esteem can be determined by positive or negative self-assessment by comparing one with others (Reilly, Rochlen, & Awad, 2014). According to Branden (1994), self-esteem is a basic human need that is important for the continuation of positive, productive functions of life, such as interpersonal relationships, workplaces, and education. High self-esteem correlates with various positive effects such as altruism, compassion, the ability to deal with change and resilience (Branden, 1994). On the opposite, low self-esteem correlated with depression (Steiger, Allemand, Robins, & Fend, 2014), addiction, and low levels of resilience and competence to overcome life difficulties (Branden, 1994).

Rosenberg, 1965, describes self-esteem as a self-related concept that refers to self-worth, feasibility, and adequacy (as cited in Gilbert & Procter, 2006). Gilbert and Procter (2006) find that low self-esteem increases an individual's vulnerability to negative mood conditions such as shame. Likewise, Wells, Glickauf-Hughes, and Jones (1999) postulate that high levels of shame are correlated with low self-esteem due to flaws and defects arising from experiences of shame that reflect low self-esteem. This correlation is very important because low self-esteem has been associated with negative mental conditions, such as depression (Johnson & O'Brien, 2013). Within a life-span, self-esteem increases during young and middle adulthood, reaching the highest point at about age 60 to 65, and declining in old age (Orth, Trzesniewski, & Robins, 2010).

Shame is generally defined as strong negative emotions characterized by perceptions of the global devaluation of oneself. Tangney and Dearing (2002) define shame as strong negative emotions in which the feeling of global self-evisceration is experienced. Shame is often generated by social events in which a personal status or feeling of rejection is sensed. Shame can refer to various aspects of the self, such as behavior or characteristics of the body, and broader identities (Hejdenberg & Andrews, 2011). In particular, the multidimensional conceptualization of shame has been posited (Andrews, Qian, & Valentine, 2002) to distinguish: 1) characteristic experiences of shyness (i.e., regarding personal habits, various styles with others, and personal skills); 2) experience shameful behavior (doing something wrong, saying something stupid, and failing in competition); and 3) bodily shame (i.e., called shame about one's physical appearance). Shame can cause severance of body image, low self-esteem, and feelings of guilt (Franzoni et al., 2013).

## Shame and Self-Esteem

Shame is a self-evaluative emotion that involves concern and attention about oneself. When shame is perceived as an emotionally painful emotion, it may have the power for self-break (Fortes & Ferreira, 2015). When individuals experience shame, the devaluation of self is perceived, and it may lower self-esteem. The frequent feeling of shame can eventually form into a trait of shame. Trait shame, in turn, involves negative feelings that are very painful and often crippling, which involve feelings of inferiority, despair, helplessness, and the eagerness to hide personal flaws (Andrews et al., 2002). Thus, it can be assumed that shame experience is closely related to fluctuations in self-esteem (Elison, Garofalo, & Velotti, 2014). Furthermore, low self-esteem can increase an individual's vulnerability to experience negative emotional states, including shame. Thus, although the direction of their association is unclear, several studies have reported a substantial relationship between low self-esteem and negative emotions, such as guilt and shame (Garofalo, Holden, Zeigler-Hill, & Vellotti, 2016).

## Demographic Dynamics in the Relationship Between Shame and Self-Esteem

As the self-concept develops, children begin to sense of self-appears at age two until they get a more stable self-concept (Lewis, 2000). During this development, at about age 3, children start to develop the capacity of self-evaluation related to differences between them and other children and understand morality and social norm (Muris & Meesters, 2014). Children and adolescents seem to have the same differences as adults in determinant factors of guilt and shame. Children, when asked about their understanding of situational determinants of guilt and shame, they state that feelings of guilt are related to violations of moral norms such as property damage or personal reproach (Ferguson, Stegge, & Damhuis, 1991). The emotion decreases with age (Williams & Bybee, 1994).

Children are believed to start experience shame only when they have reached the cognitive capacity to understand themselves as objects for reflection and have social maturity to understand and apply social scripts and rules of behavior (Emde, Johnson, & Easterbrooks, 1987; Lewis, Sullivan, Stanger, & Weiss, 1989). A cohort-sequential longitudinal study by Orth, Robins, and Soto (2010) found that shame declined from adolescence into middle adulthood, arriving at the lowest point around age 50 years, and then grew old. This variation brings impact to the self-esteem dynamics as shame requires self-evaluation, which in turn, the evaluation impacts self-esteem.

The dynamic relationship between shame and self-esteem may also be moderated by population trait: clinical and nonclinical populations. Dyer et al. (2017) conducted research comparing clinical samples (Dissociative identity disorder [DID], Complex Trauma and General Mental Health) with a healthy volunteer control group and found that the clinical groups exhibited significantly greater shame than those of nonclinical samples. These clinical traits populations are used as a moderating factor in the relationship between shame and self-esteem.

Another assumed moderating effect might derive from the various quality of the studies in meta-analysis. Quality of studies provides researchers a valid estimate of the truth of the studies (Moher et al., 1993). A standardized tool to assess the quality study classifies the study based on the characteristics of published articles. Such features are intended to estimate the precision of the findings and data in the study, where the precision is a function of systematic error and random error. It functions to classify possible causes of bias in meta-analysis outcomes as well as to describe the strengths and shortcomings of analysis in the topic of the study.

From the description above, the research questions are as follows: 1) "Does shame correlate with self-esteem?," 2) “Do age differences moderate the relationship between shame and self-esteem?" 3) “Do clinical characteristics moderate the relationship between shame and self-esteem?" and 4) "Do quality studies affect the effect size of the study?"

## Method

### Statistical Analysis

Comprehensive Meta-Analysis (Version 3.0) software (CMA; Borenstein, Hedges, Higgins, & Rothstein, 2013) is used to perform statistical analyses of publication bias, study heterogeneity, and meta-regression. The random-effects model is used to estimate the variance distribution of observed effects sizes given in participants, regions, and methods throughout the study studied (Borenstein, Hedges, Higgins, & Rothstein, 2010). When researchers decide to include a group of studies in a meta-analysis, researchers assume that research has sufficient common sense to synthesize information, but generally, there is no reason that they are “identical” in the sense that the actual effect size is the same in all studies (Konstantopoulos, 2006).

The results of each study included in this meta-analysis were quantified in the same metric, by calculating the effect size index, and then estimating effects were statistically analyzed to 1) obtain estimates of the average magnitude of the effect, 2) assess heterogeneity in-between effect estimates, and 3) looking for characteristics of research that can explain heterogeneity (Cooper, 2010). To measure heterogeneity in all studies, indicators of heterogeneity, such as *Q* and *I*-squared statistics, were calculated in this study. The Funnel plot and fail-safe *N* statistics were adopted to estimate publication bias (Egger et al., 1997). Meta-regression was used to detect the moderating effects of age, population dichotomy: clinical and nonclinical, and the quality of every study as possible sources of heterogeneity throughout this study.

### Study Search

After the research questions were formulated, the next step is to define the eligibility criteria of the study, namely the characteristics that had to be met to be included in the meta-analysis. This study follows the Preferred Reporting Items for Systematic Reviews and Meta-Analyses (PRISMA) guidelines (Liberati et al., 2009).

### Inclusion Criteria and Exclusion Criteria

We applied the following standards to screen the data found in databases:

1. English language papers: We limit studies in English, so that understanding of the content of studies is adequate.
2. Samples involving clinical or nonclinical characteristics, as well as the means of age, were retained so that it could be used as a moderator variable when heterogeneity of studies was found.
3. Measurement of shame: Shame was measured with a standard scale. Studies of shame psychometric were also coded. We ensured that the variable shame did not overlap with the concepts of shyness, embarrassment, vicarious shame, body shame, humiliation, and guilt. When found, those terms were excluded from the study.
4. Measurement of self-esteem: Self-esteem was measured using a standardized questionnaire. Any concepts related to self-esteem, such as self-concept, self-efficacy, and self-worth, were excluded from the study.
5. Study design: Selected studies are limited to quantitative studies; however, the design of the studies could vary as prospective studies, cross-sectional experiments and correlations, and psychometrics. We excluded publications that reported only qualitative data, reviews, or theoretical works.
6. Statistical information: only studies showing correlation coefficients between shame and self-esteem, whether found in pilot studies or primary studies, were selected in the analysis. Other information, such as betta weights in the regression study, was converted to the correlation coefficient.

**Figure 1:**
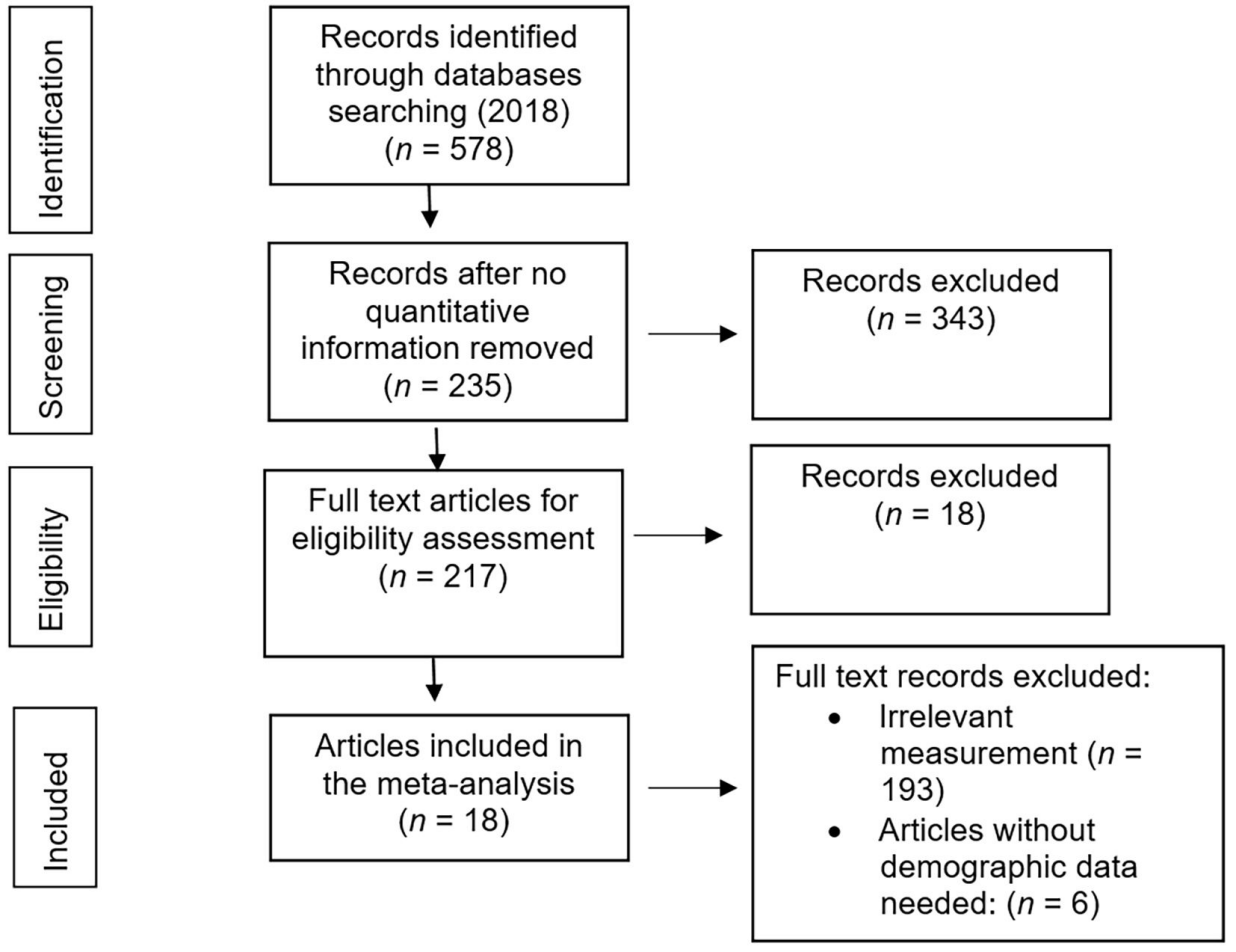
The Study Selection Process for Meta-Analysis Based on PRISMA Flow Diagram

Literature searches were conducted on the PsycINFO database, Sage Journals, Scopus, and Proquest. The keywords used in the research were: "shame," "self-esteem," "self-worth," "shame scale." The first step was to screen all 578 potential articles, as displayed in PsycInfo. Three hundred forty-three articles were excluded because quantitative data were not stated. We continued to explore the remaining 235 full articles that explicitly mentioned quantitative information in abstracts and found 217 studies that mention correlation coefficients in the results. We continued to screen for the full article and strictly selected the construct of shame and self-esteem. We excluded 193 studies that did not measure shame referred to in our study. From the selection results, we found 24 study articles that measured both shame and self-esteem and also found the effect size needed. After digging up information that could be the causes of heterogeneity in studies such as population characteristics and means of age, six studies were found that lacked both demographic information. Finally, we had 18 studies from 2002 to 2018, analyzed in the meta-analysis. (see Figure 1 for the flowchart of study selection).

Shame and esteem-related keywords such as body-shame, body esteem, collective esteem, and social esteem were excluded in the analysis. For descriptive purposes, the researcher noted the years of study, researchers, the source of the article, sample size, characteristics of the sample divided into clinical and nonclinical, and the mean age of the sample. The mean age of participants in the form of continuous data and the distribution of sample characteristics as clinical and nonclinical were used as moderators in the meta-regression analysis.

### Quality Assessments

Quality assessment of the studies in this study was adapted from the Quality Assessment and Validity Tool for Correlational Studies (Cicolini, Simonetti, & Comparcini, 2014). We employed four criteria, which are described in 13 questions to assess the design, sampling techniques, measurements, and statistical analysis of each study we study. The availability of information in the study following the question was given a score of 1 (Yes/reported), and a score of 0 (No/not measured) was given when the information needed in the study was not found. From these 13 questions, the range was 0–14 because there was one question that had a score of 2 (Yes), namely questions related to the reliability of the instruments in the study. Studies were then categorized into three groups based on total scores: low (0–4), medium (5–9), and high (10–14). Table 1 below shows the template of the quality assessment of each study.

**Table 1:** The Quality Assessment Template of Chosen Studies

Study:	First author:	
Date:	Journal:	
STUDY DESIGN	No	Yes
1. Was the study prospective?	0	1
SAMPLE		
1. Was probability sampling used?	0	1
2. Was the sample size justified?	0	1
3. Was the sample drawn for more than one site?	0	1
4. Was anonymity protected?	0	1
5. The response rate was more than 60%?	0	1
MEASUREMENT		
Shame [assess for shame correlated with self- esteem only]		
1. Was the outcome measured reliably	0	1
2. Was the outcome measured using a valid instrument?	0	1
Influence on the measure of self-esteem?		
1. Was the dependent variable measured using a valid instrument?	0	1
2. If a scale was used for measuring the dependent variable, was the internal consistency ≥ .70?	0	2
3. Was a theoretical framework used for guidance?	0	1
STATISTICAL ANALYSIS		
1. If multiple outcomes were studied, are correlation analyzed?	0	1
2. Were outliers managed?	0	1
Overall Study Validity Rating		TOTAL

*Note*. 0–4 = LO; 5–9 = MED; 10–14 = HI.

A summary of quality assessments of 18 studies that had been screened showed that 14 studies have high quality and four studies of medium quality. Four studies of medium quality were two studies of Gao, Qin, Qian, and Liu (2013), Wood, Byrne, Burke, Enache, and Morrison (2017), and Yelsma, Brown, and Ellison (2002). The quality of the study medium is due to not fulfilling random sampling criteria, participant anonymity, non-prospective study designs, and sampling from various sites. Fourteen studies reviewed were of high quality (Feiring, Taska, & Chen, 2002; Goss, 2013; Greene & Britton, 2013; Legate, Weinstein, Ryan, DeHaan, & Ryan, 2019; Passanisi, Gervasi, Madonia, Guzzo, & Greco, 2015; Pilarska, 2018; Reilly et al., 2014; Simonds et al., 2016; Velotti, Garofalo, Bottazzi, & Caretti, 2017; Ward, 2014; Woodward, McIlwain, & Mond, 2019; Zhou, Wang, & Yi, 2018). Of all studies, only a study by Feiring et al. (2002) conducted a retrospective study even though the selection of samples was not random. The majority of studies in this meta-analysis did not conduct random sampling and outlier handling in the analysis. A moderation analysis of the study quality classification was carried out to see its effect on the overall effect size. The moderation analysis was carried out together with age, characteristics of the samples, and quality study by meta-regression. Table 2 summarizes the quality assessment of 18 studies included.

**Table 2:** Summary of Quality Assessment of Each Study

Standard	Number of studies	
	Yes	No
Design		
Prospective study	1	17
Sample		
Probability sampling	0	18
Proper sample size	18	0
Sample drawn for more than one site	13	5
Anonymity assurance	11	7
Response rate > 60%	18	0
Measurement		
Reliable measures of outcomes	18	0
Valid measure of self-esteem	18	0
Valid measure of shame	18	0
Statistical Analysis		
Correlation analysis when multiple effect studied	17	1
Management of outliers addressed	0	18

## Results

In this study, the effects size index used is the correlation coefficient. When the correlation coefficient is used as a measurable effect, both Hedges and Olkin and Rosenthal and Rubin recommend the transformation of this effect size to a standard normal metric (using the *r*-to-Fisher *Z* transformation). The following Table 3 shows the distribution of research data studied, supplemented by information on the correlation coefficients that have been transformed into Fisher's *Z* along with their standard errors, variances, *Z* values, and *p*-values.

**Table 3:** Summary of Studies in the Meta-Analysis

Study no.	Study name	*N*	Effect direction	*r*	*SE*	Variance	Fisher's *Z*	95% CI	*SE*	Variance	*M*_Age_	Population characteristic
1	Feiring et al. (2002)	137	Negative	−.17	.08	0.007	−0.17	[−0.495,−0.258]	0.09	0.007	11.50	Clinical
2	Gao et al. (2013)	277	Negative	−.36	.05	0.003	−0.38	[−0.582,−0.103]	0.06	0.004	22.57	Nonclinical
3	Gao et al. (2013)	70	Negative	−.33	.11	0.012	−0.34	[−0.495,−0.258]	0.12	0.015	20.54	Nonclinical
4	Goss (2013)	179	Negative	−.56	.05	0.003	−0.63	[−0.620,−0.325]	0.07	0.006	27.20	Clinical
5	Goss (2013)	180	Negative	−.43	.06	0.004	−0.47	[−0.754,−0.601]	0.07	0.006	27.20	Clinical
6	Greene and Britton (2013)	657	Negative	−.59	.02	0.001	−0.68	[−0.835,−0.402]	0.04	0.002	34.89	Nonclinical
7	Legate et al. (2019)	484	Negative	−.63	.03	0.000	−0.74	[−0.784,−0.503]	0.05	0.002	28.40	Clinical
8	Passanisi et al. (2015)	209	Negative	−.57	.05	0.002	−0.65	[−0.784,−0.511]	0.07	0.005	21.66	Nonclinical
9	Pilarska (2018)	357	Negative	−.33	.05	0.002	−0.34	[−0.341,−0.002]	0.05	0.003	21.19	Nonclinical
10	Reilly et al. (2014)	145	Negative	−.55	.06	0.003	−0.62	[−0.783,−0.454]	0.08	0.007	26.01	Nonclinical
11	Simonds et al. (2016)	85	Negative	−.55	.08	0.006	−0.62	[−0.630,−0.339]	0.11	0.012	13.55	Nonclinical
12	Velotti et al. (2017) ^a^	251	Negative	−.51	.05	0.002	−0.56	[−0.687,−0.438]	0.06	0.004	28.50	Nonclinical
13	Velotti et al. (2017) ^b^	129	Negative	−.42	.07	0.005	−0.45	[−0.622,−0.273]	0.09	0.008	31.00	Nonclinical
14	Ward (2014)	115	Negative	−.68	.05	0.003	−0.83	[−1.014,−0.644]	0.09	0.009	24.13	Nonclinical
15	Wood et al. (2017)	79	Negative	−.98	.05	0.000	−0.30	[−2.522,−2.073]	0.11	0.013	36.49	Clinical
16	Woodward et al. (2019)	403	Negative	−.83	.01	0.000	−0.19	[−0.985,−0.789]	0.05	0.003	23.90	Nonclinical
17	Yelsma et al. (2002)	185	Negative	−.45	.06	0.003	−0.48	[−0.447,−0.239]	0.07	0.005	21.00	Nonclinical
18	Zhou et al. (2018)	263	Negative	−.48	.05	0.002	−0.52	[−0.645,−0.401]	0.06	0.004	34.60	Nonclinical

^a^Female participants. ^b^Male participants.

### Heterogeneity Study

Heterogeneity testing of all studies is summarized in Table 4. *I*^2^ statistics for heterogeneity was 95.09 (95.09%), *p* < .001, which resulted in the acceptance of alternative hypotheses and showed significant heterogeneity in the studies taken. *I*^2^ shows the amount of variability that cannot be explained by chance. In other words, *I*^2^ index explains the percentage of variability estimate (95.09%) in results across studies that is due to real differences and not due to chance. Also, *Q* values higher than *df* indicates heterogeneity.

**Table 4:** Heterogeneity Test Across Studies

*I* ^2^	*Q*	*df*	*p*
95.09	346.44	17	.000

**Figure 2:**
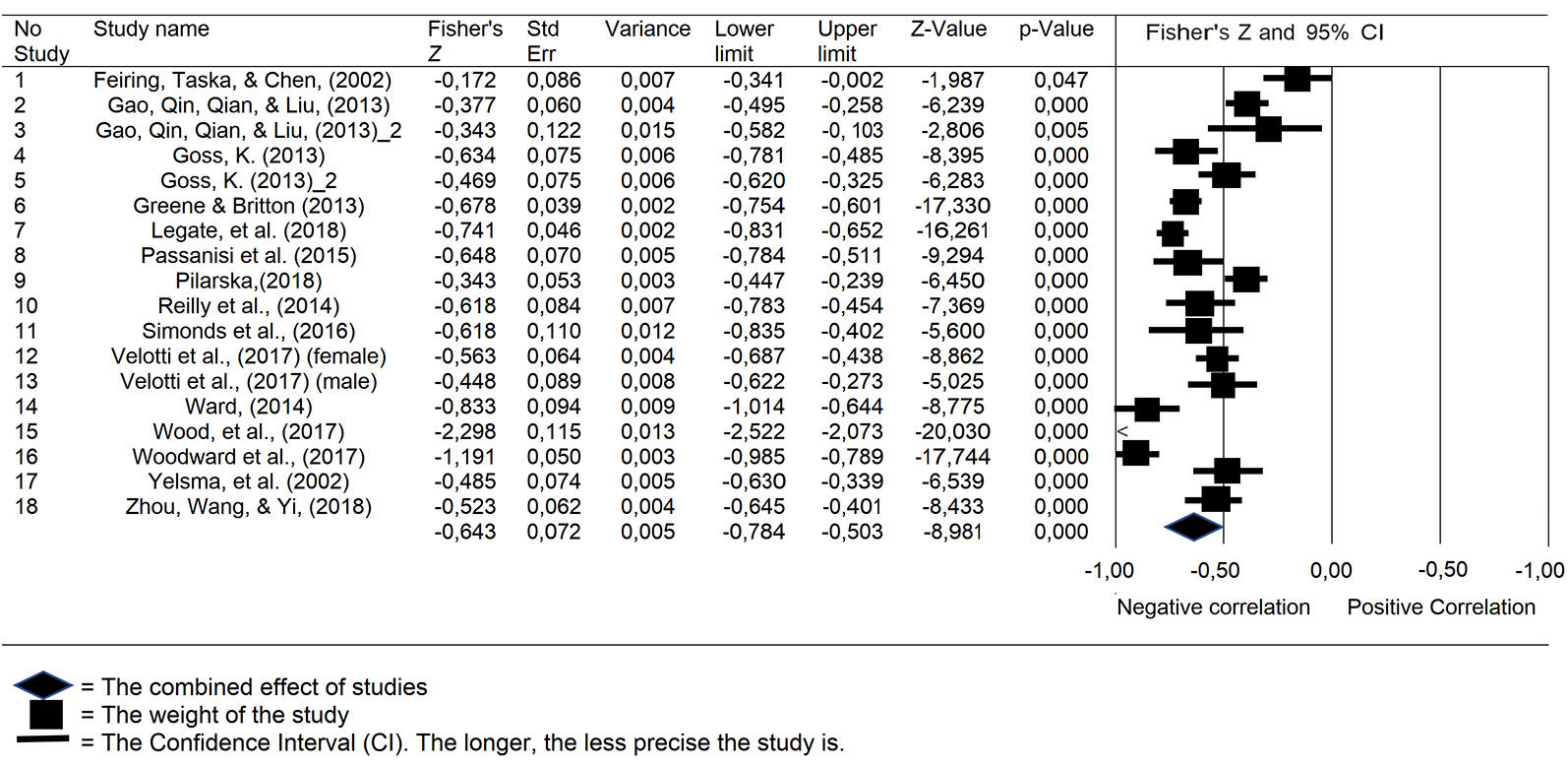
The Summary of Studies in the Meta-Analysis

Figure 2 below summarizes the results of the meta-analysis with 95% Confidence Interval (CI). The analysis carried out in this study was based on the random-effects model due to the non-homogeneous characteristics of the population. In Feiring's study, the horizontal line/CI almost touched the value of 0 so that the *p*-value (.047) was close to .05. The forest plot shows that study 2 by Gao et al. (2013) and Simonds et al. (2016) have wide plot lines. The plotline indicates a wider CI, which means that the study has low precision. The overall summary information at the 95% confidence interval shows that in the correlation study between shame and self-esteem, the random effect size is **−**.643 (moderate effect), with *Z*-value = **−**8.981 and *p* < .001. Based on the effect size value with *p* < .001, the alternative hypothesis cannot be rejected so that there is a negative correlational relationship between shame and self-esteem.

### Funnel Plots

One other mechanism for displaying the relationship between study size and effect size is Funnel Plots. In this study, the use of standard errors (rather than sample size or variance) on the Y-axis has the advantage of spreading points at the bottom of the scale, where smaller studies are plotted. This can make it easier to identify patterns of asymmetry (Borenstein, Hedges, Higgins, & Rothstein, 2009). Funnel Plots is a spread of effect size on a measure of study accuracy. This description provides information support in the meta-analysis, mainly related to the heterogeneity of studies (Stuck et al., 1998).

**Figure 3:**
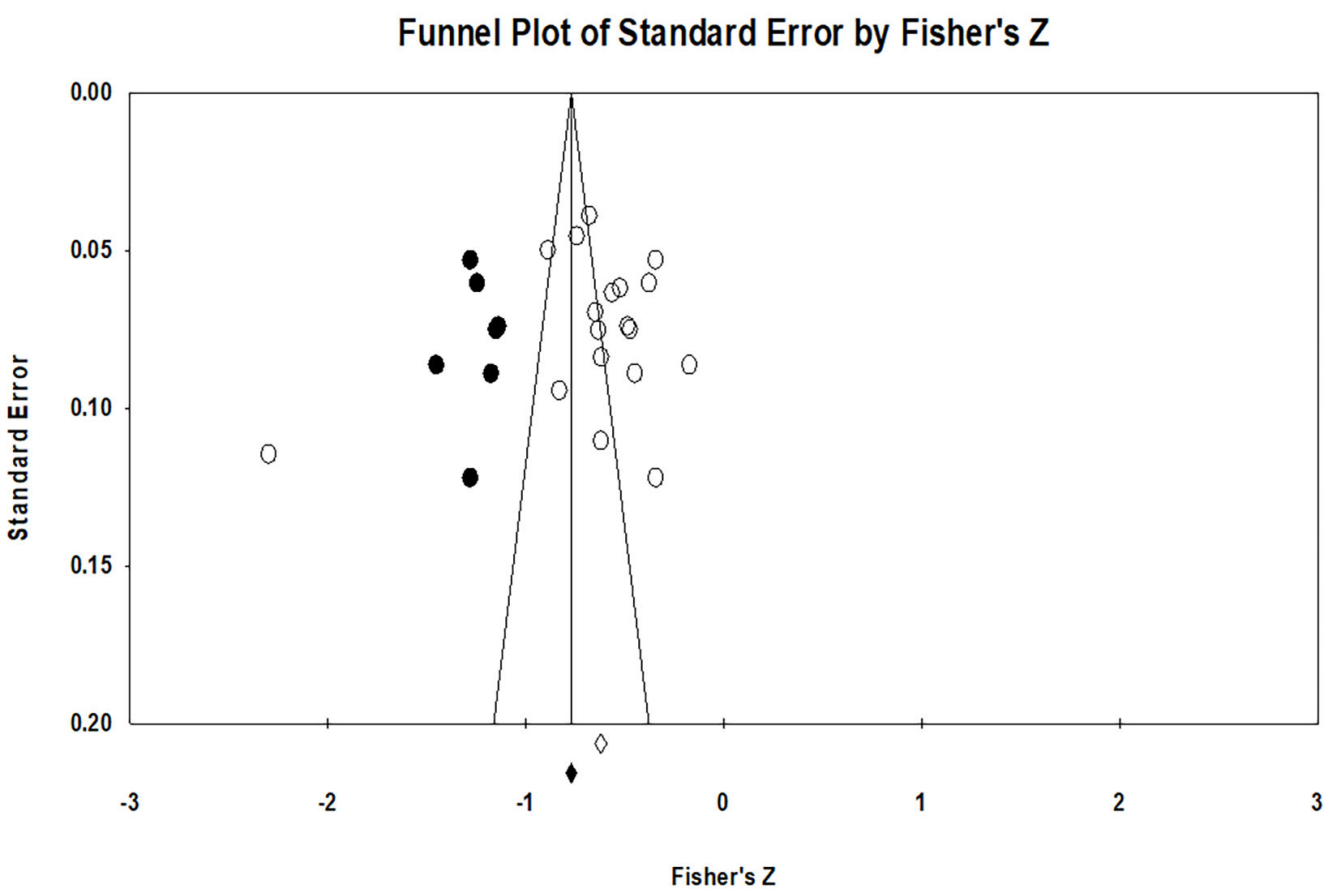
Funnel Plots of the Observed and Imputed Studies

Based on Funnel Plots in Figure 3, it appears that the distribution of effects on the standard error forms a "funnel," giving the impression that there are no biases in the analyzed studies, no asymmetrical plot.

The majority of studies with large effects appear towards the top of the graph and tend to cluster near the mean effect size. More small-effect research appears to the bottom of the graph, and because there are more sampling variations in the estimation of effect sizes in studies with small effects, this study will be spread over a range of values.

With no publication bias in these studies, the lower part of the plot does not show a higher concentration of studies on one side of the mean than the other. This graph reflects the fact that studies that have smaller effects (which appear downward) are more likely to be published if they have a greater effect than the mean effect, which makes them more likely to meet the criteria for significant statistics (Hunter et al., 2014).

### Fail-Safe *N*

The fail-safe *N* related to publication bias in this study uses Orwin, 1983, approach (as cited in Borenstein et al., 2009) as summarized in Table 5. The Orwin approach allows researchers to determine how many studies are missing, which will bring the overall effect to a specified level other than zero. Therefore, researchers can choose a value that will represent the smallest influence that is considered important and substantive, and ask how many missing studies are needed to bring the summary effect below this point.

**Table 5:** The Orwin's Fail-Safe N Test

Orwin's fail-safe *N*	Criterion
Fisher's *Z* in observed studies	−0.621
The criterion for a trivial Fisher's *Z*	−0.600
Mean Fisher's *Z* in missing studies	0.000
Number of missing studies to bring Fisher's *Z* over −0.650	1.000

The mean Fisher's *Z* in the new study (which is missing) can be a value other than zero, which in this study, was set at 0.00001. Also, the value of the criteria used is the effect size *Z* instead of the *p*-value. This means that the Orwin fail-safe *N* is the number of missing studies, which when added to the analysis, will bring the combined *Z* value above the specified threshold (currently the upper limit is set at, 0.600). The fail-safe *N* Orwin numbers obtained is 1. This result means that we need to find 1 study with the mean Fisher's *Z* value of 0.648 to bring the combined *Z* value above the value 0.650.

### Bagg and Mazumdar's Rank Correlation

The rank correlation test uses the Begg and Mazumdar tests (Begg & Mazumdar, 1994), which involve correlations between effect sizes rank and variances rank, respectively. The result of the analysis shows a value of *p* = .879, indicating the acceptance of the null hypothesis and showing no publication bias. In this case, Kendall's value *b* is **−**0.026, with *p*-value 1-tailed (recommended) of .439 or *p* two-tailed value of .444 based on normal estimated continuity correction, as shown in Table 6. The estimated value of Kendall's tau rank correlation coefficient shows that the observed outcomes and the corresponding sampling variances are not highly correlated. This finding means that a very low correlation would indicate that the funnel plot is symmetric, which may not show a result of publication bias.

**Table 6:** Rank Correlation Test for Funnel Plot Asymmetry

Kendall's Tau	*Z* for Tau	*p*
−.03	0.15	.439 (one-tailed)
		.879 (two-tailed)

### Egger's Regression Intercept

Egger shows that the bias assessment is based on precision (the opposite of the standard error) to predict standardized effects (effect size divided by standard error). In this equation, the measure of the effect is captured by the slope of the regression line (*B*_1_), while it can be captured by the intercept (*B*_0_). In this study, the intercept (*B*_0_) was −0.946, 95% CI[−8.581, 6.688], with *t* = 0.263, *df* = 16. The *p* 1-tailed value (recommended) was .398, and the *p* 2-tailed value is .796 indicating no evidence of publication bias.

### Duval and Tweedie's Trim and Fill

Based on Funnel Plots analysis, the observed and imputed plots were detected that more studies were on the right side than those on the left side. Therefore, the assumption that arises is that missing studies have occurred on the left side of axis X. In Figure 3, observed studies are described as open (colorless) circles, while six imputed studies are represented by black circles.

If the meta-analysis has captured all relevant studies, it is expected that the funnel plots will be symmetrical. That is, we would expect research to be spread evenly on both sides of the overall effect.

Duval and Tweedie went more advanced by a method that allowed us to link these missing studies. That is, the researcher determines where the missing study tends to "disappear," then adds it to the analysis, and then recalculates the combined effect. This method is known as Trim and Fill (Duval & Tweedie, 2000).

If we refer to Figure 3, an asymmetrical study of the right side is trimmed to find an unbiased effect (in an iterative procedure) and then fill the plot by re-entering the study trimmed on the left side of the mean effect. This program looks for missing studies based on the random-effects model and looking for studies that are lost only to the left side of the mean effect. This method shows that there are seven missing studies. Based on the random effect model, the estimated points at the 95% confidence interval for the combined study are **−**0.643 (**−**0.784, **−**0.502). Using Trim and Fill, the estimated imputed points are **−**0.812 (**−**0.962, **−**0.663). See Table 7 for Duval and Tweedie's Trim and Fill output.

**Table 7:** Duval and Tweedie's Trim and Fill

Value	Studies trimmed	Point estimate	*LL*	*UL*	*Q*
Observed values		−0.643	−0.784	−0.502	346.444
Adjusted values	7	−0.812	−0.962	−0.663	741.736

### Age, Characteristics of Samples, and Quality of Studies as Moderators

After heterogeneity of the studies is detected, the next step is to identify the variables and characteristics which cause heterogeneity. Meta-regression analysis is used to estimate the parameter effects with minimum variance. In this study, the age, population characteristics, and study quality are considered as covariates between shame and self-esteem.

The age, clinical/nonclinical characteristics, and high and low study qualities moderating effect tests are based on the random-effects model with the restricted maximum likelihood model (REML) estimation method. Based on the analysis using a meta-regression test, it can be explained that the regression coefficient for age is equal to **−**0.03, which means that every one degree of age equals a decrease in the effect size of 0.03. The *p* = .02 shows the variable age functions as a moderator in the relationship between shame and self-esteem. Thus, it can be concluded that age moderates the relationship between shame and self-esteem because age is significantly related to effect size. Differences in sample characteristics based on clinical and nonclinical groups do not have a moderating effect on the relationship between shame and self-esteem (*p* = .232). The quality of studies that are categorized into high and moderate-quality does not moderate the relationship between shame and self-esteem (*p* = .184). Study quality, age, and sample characteristics simultaneously affect the effect size (*p* = .03). The summary of the meta-regression is shown in Table 8.

**Table 8:** Moderating Testing (Random Effects, REML Method)

Covariate	Coefficient	*SE*	95% CI		*Z*	*p* (2-sided)
			*LL*	*UL*		
Intercept	−0.007	0.38	−0.75	0.73	−0.02	.985
Clinical characteristics	−0.229	0.19	−0.61	0.15	−1.20	.232
Study quality	0.276	0.21	−0.13	0.68	1.33	.184
Age	−0.031	0.01	−0.06	−0.01	−2.42	.015

*Note*. Simultaneous test: *Q* = 9.32, *df* = 3, *p* = .03.

**Figure 4:**
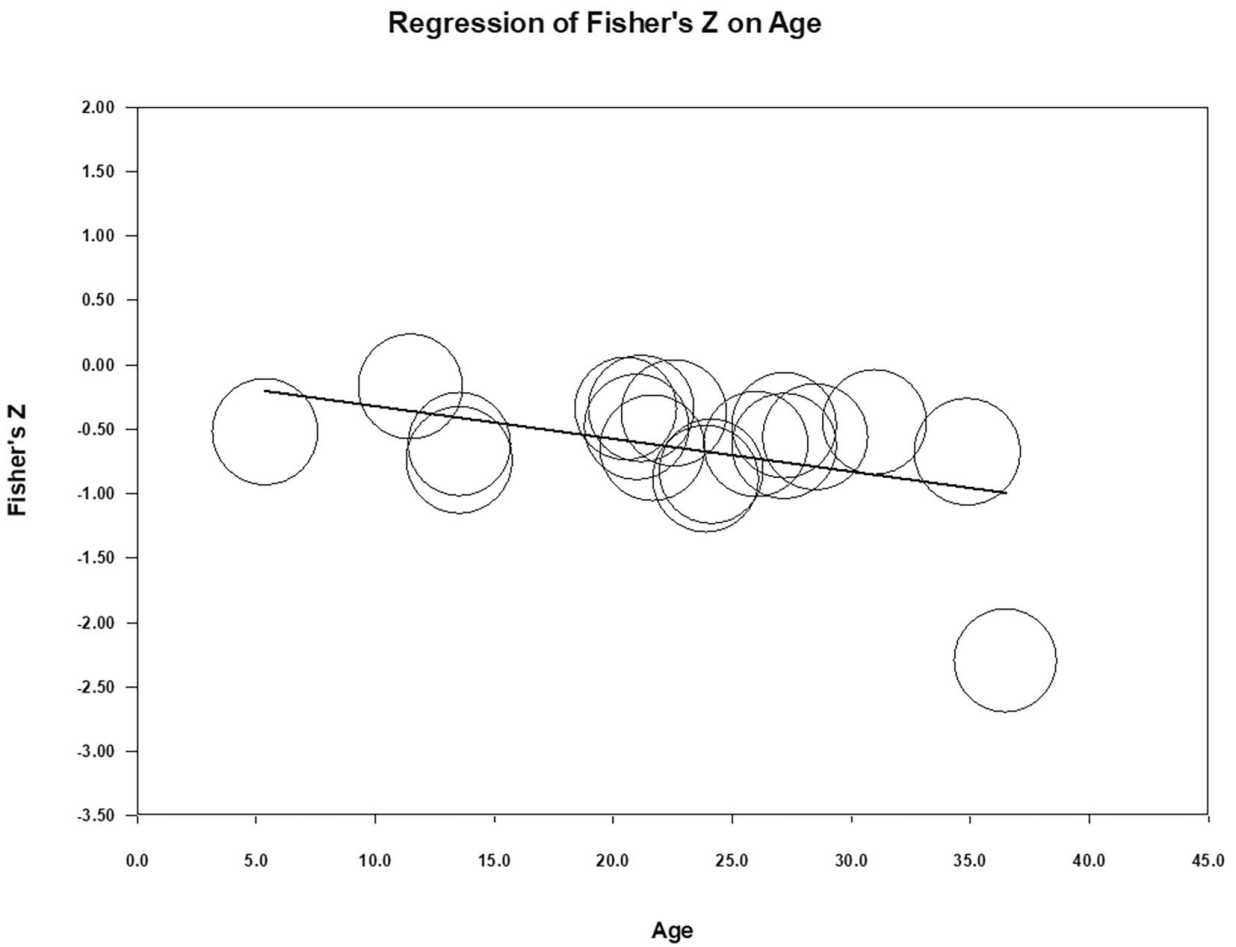
Regression Plot of Age as a Moderator

Apart from the above calculations, a scatter plot can also explain the pattern of the relationship between age as a moderator and the observed effect size. The scatter diagram below illustrates that there is a clear relationship between age as a moderator and the observed effect size. It can be concluded that as age increases, the effect size moves away from 0. This means that the relationship between shame and self-esteem (when other covariates are controlled) gets stronger as we age. Figure 4 shows the regression plot of age as a moderator.

## Discussion

The meta-analysis results displayed a negative correlation between shame and self-esteem with effect size is different from zero (*r* = **−**.643, *p* < .001). The random-effects model analysis shows the mean of the distribution of the true effects is 0.643. According to Cohen's classification, it is classified as a large effect size. This finding supports the research hypothesis that there is a relationship between shame and self-esteem.

Shame, as self-conscious emotion, deals with negative, global, and stable evaluations of the self (Tangney & Dearing, 2002), and it brings impact to the fluctuation of self-esteem. When a person perceives himself as "a bad person," their self-esteem decreases. Usually, feelings of shame happen due to a condition where the personal self is devalued, such as a bad performance socially assessed. Poor performance leads to greater reactions of psychological states indicating a danger to the social self, namely a decline in social self-esteem and an increase in shame (Gruenewald, Kemeny, Aziz, & Fahey, 2004).

Many shameful experiences can eventually crystallize into a trait-like proneness of shame. Trait shame, in turn, includes an especially painful and often disabling, adverse sensation involving a sense of inferiority, hopelessness, and helplessness, as well as a willingness to conceal private failure (Andrews et al., 2002). Also, shame experiences have been suggested to be closely linked to fluctuations in self-esteem, and many shame experiences might be conceptually linked to chronically low self-esteem rates (Elison et al., 2014).

This study shows that based on publication bias testing with information from *funnel plots*, *fail-safe N*, *Bagg and Mazumdar rank correlations*, *Egger's Regression Intercept,* and *Duval and Tweedie's Trim and Fill* no publication bias was found.

To explain what factors causing heterogeneity of the study, the thing that researchers can do is simply enter the mean age of the samples to be analyzed as moderators in the relationship between shame and self-esteem. Analysis with meta-regression showed that the age of various participants moderated the effect size of the relationship of shame and self-esteem. This result shows that the selection of random effect models as the basis of the meta-analysis in this study is appropriate. However, the study qualities and clinical characteristics of the sample did not moderate the effect size.

In a prospective study, De Rubeis and Hollenstein (2009) found that, during early adolescence, shame slightly decreased over a period of 1 year. Similarly, self-esteem follows a quadratic life-span trajectory, increasing during young and middle adulthood, peaking at about 60 to 65 years of age, and declining in old age (Orth, Trzesniewski, et al., 2010). This age dynamics influence the quality of shame and self-esteem relationship.

In the context of the study sample with clinical and nonclinical characteristics, no moderating effect was found. This finding indicates that the dynamics of shame and self-esteem in both characteristics of the research sample are similar. The clinical samples in this study were various, involving those with an eating disorder (Goss, 2013), schizoaffective disorder (Wood et al., 2017), sexual abuse (Feiring et al., 2002), and lesbian, gay, or bisexual (LGB; Legate et al., 2019). These different clinical characteristics may interfere with the moderation effect when comparing to nonclinical samples.

In the process of selecting a study, the initial screening has been done so that the variables of shame that are analyzed only involve shame based on self-evaluation in an embarrassing event. Thus, the various measures of shame that are not included in this study include body-shame and trait shame. However, this study still finds heterogeneity in the studies studied. When sensitivity analysis is carried out by looking at relative weight images, there are no different relative weights from the studies analyzed, so that study ejection is not carried out from the analysis. From this, it can be concluded that the occurrence of heterogeneity is not caused by sampling error.
